# High-Dialysate-Glucose-Induced Oxidative Stress and Mitochondrial-Mediated Apoptosis in Human Peritoneal Mesothelial Cells

**DOI:** 10.1155/2014/642793

**Published:** 2014-05-05

**Authors:** Kuan-Yu Hung, Shin-Yun Liu, Te-Cheng Yang, Tien-Ling Liao, Shu-Huei Kao

**Affiliations:** ^1^Department of Internal Medicine, National Taiwan University Hospital, College of Medicine, National Taiwan University, Taipei 106, Taiwan; ^2^School of Medical Laboratory Science and Biotechnology, College of Medical Science and Technology, Taipei Medical University, Taipei 110, Taiwan; ^3^Division of Nephrology, Department of Internal Medicine, Kuan-Tien General Hospital, Taichung 433, Taiwan; ^4^Graduate Institute of Medical Science, College of Medicine, Taipei Medical University, Taipei 110, Taiwan

## Abstract

Human peritoneal mesothelial cells (HPMCs) are a critical component of the peritoneal membrane and play a pivotal role in dialysis adequacy. Loss of HPMCs can contribute to complications in peritoneal dialysis. Compelling evidence has shown that high-dialysate glucose is a key factor causing functional changes and cell death in HPMCs. We investigated the mechanism of HPMC apoptosis induced by high-dialysate glucose, particularly the role of mitochondria in the maintenance of HPMCs. HPMCs were incubated at glucose concentrations of 5 mM, 84 mM, 138 mM, and 236 mM. Additionally, N-acetylcysteine (NAC) was used as an antioxidant to clarify the mechanism of high-dialysate-glucose-induced apoptosis. Exposing HPMCs to high-dialysate glucose resulted in substantial apoptosis with cytochrome c release, followed by caspase activation and poly(ADP-ribose) polymerase cleavage. High-dialysate glucose induced excessive reactive oxygen species production and lipid peroxidation as well as oxidative damage to DNA. Mitochondrial fragmentation, multiple mitochondrial DNA deletions, and dissipation of the mitochondrial membrane potential were also observed. The mitochondrial dysfunction and cell death were suppressed using NAC. These results indicated that mitochondrial dysfunction is one of the main causes of high-dialysate-glucose-induced HPMC apoptosis.

## 1. Introduction

Peritoneal dialysis (PD) is an effective mode of renal replacement therapy for patients with end-stage renal disease [1, 2]. The success of PD depends on the structural and functional integrity of the peritoneal membrane. Human peritoneal mesothelial cells (HPMCs) are a pivotal component of the peritoneal membrane [3]. During chronic PD, HPMCs are repeatedly exposed to a nonphysiologic environment. However, the long-term use of conventional PD fluids causes histological and functional alterations of the peritoneum, resulting in HPMC apoptosis, extracellular matrix (ECM) accumulation, peritoneal fibrosis, and ultimately peritoneal function failure [4, 5]. Identifying the mechanism underlying the adverse effects of PD fluids and determining potential therapeutic strategies for preserving a functional peritoneum in the long term are crucial.

The currently used PD fluids are glucose-based solutions. Glucose is an effective osmotic agent and readily metabolized at high concentrations. Both in vivo and in vitro studies have shown no significant effect of high mannitol on HPMC apoptosis, indicating that high-dialysate glucose induces cell death unrelated to hyperosmolarity [6]. However, evidence has shown that high-dialysate glucose is a key factor causing peritoneal membrane dysfunction [7]. High glucose promotes apoptosis in several cell types, including vascular endothelial cells and renal mesangial cells [8, 9]. Long-term exposure to high-dialysate glucose in PD fluid might contribute to HPMC apoptosis.

Mitochondria not only create energy for cells but also play an integral role in apoptosis [10]. As the centre organelle of the intrinsic apoptotic pathways, mitochondria are the reservoir of proapoptotic factors including cytochrome c, Smac/DIABLO, EndoG, and HtrA2/Omi. The impairment of mitochondrial function causes cell death by compromising ATP production, calcium homeostasis, and increasing oxidative stress. These stimuli induce the mitochondrial permeability transition pore opening, mitochondrial membrane potential dissipation, and the release of proapoptotic proteins. After releasing from mitochondria, cytochrome c binds and activates Apaf-1 and procaspase-9 to form an apoptosome that results in caspase activation and subsequent cleavage of structural and regulatory proteins in cytoplasm and nucleus, leading to cell death [11]. Therefore, the role of mitochondria in the process of HPMC apoptosis has become a research focus.

According to diabetes-related studies, the initial reaction of the cells upon exposure to high glucose is an increased production of reactive oxygen species (ROS). ROS form as by-products of oxidative phosphorylation in mitochondria. Mitochondrial overproduction of ROS in hyperglycemia has been postulated to cause redox imbalance, oxidative insults, mitochondrial dysfunction, and cell death [12, 13].

Therefore, mitochondria are rationally suspected to be a causal link between high-dialysate glucose and HPMC apoptosis. Approaches to reducing HPMC damage that interfere with apoptosis should be considered.

In this study, we characterized the molecular mechanism of high-dialysate-glucose-induced apoptosis, focusing on mitochondria in the apoptotic pathway. This study provides a basis for further development of a potential therapeutic strategy for protecting the integrity of the peritoneum membrane in long-term PD.

## 2. Materials and Methods

### 2.1. Reagents

Fetal calf serum (FCS) was obtained from Biochrome KG (Berlin, Germany). Culture flasks and plates were purchased from Corning (Corning, NY, USA) and precoated with 1.6 *μ*g/cm^2^ of Vitrogen 100 (Celtrix Lab, Palo Alto, CA, USA) before cell seeding. M199 media, trypsin-ethylenediaminetetraacetic acid, glutamine, and trypan blue were obtained from Gibco (Grand Island, NY, USA). Bovine serum albumin (BSA), N-acetylcysteine (NAC), 3-[4,5-dimethylthiazol-2-yl]-2,5-diphenyltetrazolium bromide (MTT), and other tissue culture reagents were purchased from Sigma (St. Louis, MO, USA). An ATP assay kit, CM-H_2_DCFDA, MitoSox, MitoTracker Red, and JC-1 were purchased from Molecular Probes (Eugene, OR, USA). Annexin V-fluorescein isothiocyanate (FITC) and propidium iodide (PI) (TACS Annexin V-FITC Apoptosis Detection Kits) were purchased from R&D Systems (Minneapolis, MN, USA).

### 2.2. Preparation of Human Peritoneal Mesothelial Cells

The primary HPMC culture was established as described previously [9]. Briefly, the surgically removed human omentum was washed 3 times with phosphate buffered saline (PBS) and then digested with trypsin-EDTA (Gibco) for 15 min. After centrifugation, the cell pellet was washed with an M199 culture medium and then seeded into a Vitrogen-coated flask. An M199 medium containing 100 mg/dL of glucose and 20% FCS, penicillin (100 U/mL), streptomycin (100 *μ*g/mL), and insulin (30 *μ*g/mL) was used. The cells became confluent and were subcultured with a medium containing 10% FCS. All in vitro experiments were performed between passages 2 and 3. This study was performed according to the tenets of the Declaration of Helsinki for research involving human subjects. The protocol was approved by the National Science Council of Taiwan and the Institutional Review Board and Ethics Committee of National Taiwan University Hospital. After informed patient consent was received, human omentums were obtained from abdominal surgical procedures for elective gastric cancer resection.

Dianeal solution (Baxter, Norfolk, UK) is currently used for PD in Taiwan and contains 1.5%, 2.5%, and 4.25% glucose, equal to glucose concentrations of 84 mM, 138 mM, and 236 mM, respectively. HPMCs were exposed to high-dialysate glucose at the various concentrations for 24 h and various evaluations were then performed. NAC was added to the culture medium for 1 h before exposure to high-dialysate glucose stimulation to evaluate the antioxidant effect of NAC.

### 2.3. Cell Viability Determination through Trypan Blue Exclusion

Various concentrations of high-dialysate glucose were added to the HPMCs until final concentrations of 84 mM, 138 mM, and 236 mM were reached, and 5 mM glucose was used in a control group. Trypan blue exclusion was used to determine cell viability. Briefly, approximately 10 *μ*L of cell suspensions in PBS was mixed with 40 *μ*L of trypan blue (Gibco, Grand Island, NY, USA), and the numbers of stained (dead cells) and unstained cells (live cells) were counted using a hemocytometer.

### 2.4. Flow Cytometry Analysis of Human Peritoneal Mesothelial Cell Apoptosis

Detection of cell apoptosis was performed using both annexin V-FITC and PI staining. Aliquots of 1 × 10^6^ cells were gently stained with fluorescent probes and annexin V-FITC and PI, in the dark for 15 min at room temperature. After being stained, the cells were washed with PBS, and a flow cytometric analysis was conducted. All analyses were performed using FACScan (Becton Dickson, San Jose, CA, USA). A minimum of 30** **000 cells per sample were analyzed.

### 2.5. Western Blot Analysis of the Apoptosis-Related Protein

HPMCs were lysed in a RIPA buffer. Equal amounts of a protein were electrophoresed on an acrylamide gel and transferred to polyvinylidene difluoride membranes (Millipore, Billerica, MA, USA) by using a transblot chamber with 5% BSA in a Tris saline Tween buffer (20 mM Tris, pH 7.4, 0.5 M NaCl, 0.05% Tween 20). Western blots were incubated at 4°C overnight with the appropriate primary antibodies for cytochrome c, caspase-9, caspase-3, poly(ADP-ribose) polymerase (PARP), *α*-tubulin, cytochrome c oxidase IV (COXIV), and *β*-actin (Santa Cruz Biotechnology, Santa Cruz, CA, USA). The membranes were washed with PBS-Tween-20 and incubated for 1 h with secondary antibodies (anti-rabbit-IgG-conjugated horseradish peroxidase) (Cell Signaling Technologies, Beverly, MA, USA) and subsequently subjected to enhanced chemiluminescence detection (Amersham, Uppsala, Sweden).

### 2.6. Flow Cytometry Analysis of Reactive Oxygen Species Generation, Lipid Peroxides, 8-Hydroxy-2′-deoxyguanosine, and Mitochondrial Superoxides

To quantify intracellular ROS generation in HPMCs, a modified flow-cytometry-based analysis was performed. Aliquots of 1 × 10^6^ cells were gently stained with fluorescent probes in the dark for 15 min at room temperature. After being stained, the cells were washed with PBS, and flow cytometric analysis was conducted. Intracellular ROS were stained with CM-H_2_DCFDA (Molecular Probes, Eugene, OR, USA) and measured using a 490–500 nm wavelength for excitation and a 525 nm wavelength for emission. Lipid peroxidation was detected using C11-BODIPY^581/591^ green fluorescence (molecular probes). The fluorescence excitation and emission of the C11-BODIPY^581/591^ shifted from 581/591 nm to 490/510 nm after oxidation.

To measure 8-hydroxy-2′-deoxyguanosine (8-OHdG), aliquots of 1 × 10^6^ cells were harvested, fixed with 4% paraformaldehyde, and immunoreacted with the anti-8-OHdG antibody. After fixation, the cells were incubated for 1 h at room temperature with 2% BSA to prevent the nonspecific binding of antibodies and then incubated for 30 min with 10 *μ*g/mL of the anti-8-OHdG mouse monoclonal antibody (Genesis Biotech, Taipei, Taiwan). The cells were subsequently washed with PBS and treated with FITC-conjugated AffiniPure goat anti-mouse IgG (ImmunoResearch, West Grove, PA, USA).

Mitochondrial superoxide levels were measured by performing MitoSox Red staining. MitoSox Red is a highly selective fluorescent probe dye for mitochondrial superoxides (O_2_^∙^^−^), exhibiting an excitation at 510 nm and an emission at 580 nm.

### 2.7. High-Performance Liquid Chromatography Detection of Lipid Peroxides

Lipid peroxide content was determined in the form of malondialdehyde (MDA) as a thiobarbituric acid reactive substance (TBARS). In brief, an aliquot of cell lysates was pipetted into a test tube containing 0.6 mL of 0.44 M phosphoric acid. After mixing, 0.2 mL of a 42 mM thiobarbituric acid solution was placed in a 95°C dry bath for 1 h. The samples were then neutralized with 1 N NaOH in methanol before a high-performance liquid chromatography (HPLC) analysis was conducted. An aliquot of 20 *μ*L of a supernatant was injected into a C_18_ column (4.6 × 250 mm, with a particle size of 5 *μ*m) by using a Jasco PU-980 pump (Easton, MD, USA) with a solvent system composed of methanol and a 50 mM phosphate buffer (pH 6.8) (4 : 6, v/v) at a flow rate of 1 mL/min. The eluent was monitored by fluorescence detector at 525 nm excitation wavelength and 550 nm emission wavelength.

### 2.8. Confocal Microscope Analysis of Mitochondrial Morphology

To visualize mitochondrial morphology, cells were stained with MitoTracker Red for 10 min, mounted using fluoromount, and examined using a confocal fluorescence microscope (TCS SP5; Leica Microsystems CMS GmbH, Mannheim, Germany). Confocal fluorescent images were captured using the Leica SP5 confocal microscope fitted with an Apochromat 63 × 1.4 NA immersion objective, with excitation at 579 nm and emission at 599 nm. Images were acquired using a camera (DFC 350 FX 1.8.0; Leica) and LAS AF software (Leica).

### 2.9. Flow Cytometry Analysis of Mitochondrial Membrane Potential

Mitochondrial activity depends on active membrane potential. The mitochondrial membrane potential dye JC-1 acts as a marker of mitochondrial activity, existing as a green fluorescent monomer at low membrane potentials and as orange fluorescent aggregates at high membrane potentials. In brief, cells were collected and incubated for 10 min with 5 *μ*M JC-1 (molecular probes) at 37°C. A minimum of 30** **000 cells per sample were analyzed. The data were quantified using the LYSYS II software program (Becton Dickson).

### 2.10. Statistical Analysis

Data are expressed as mean ± standard error mean (SEM). Comparison between 2 values was performed using an unpaired Student's *t*-test. For multiple comparisons among different groups of data, the significant differences were determined using the one-way ANOVA method. Significance was defined at *P* < 0.05.

## 3. Results

### 3.1. High-Dialysate-Glucose-Induced Human Peritoneal Mesothelial Cell Apoptosis through Cytochrome c Release and Caspase Cascade Activation

**Figure 1:**
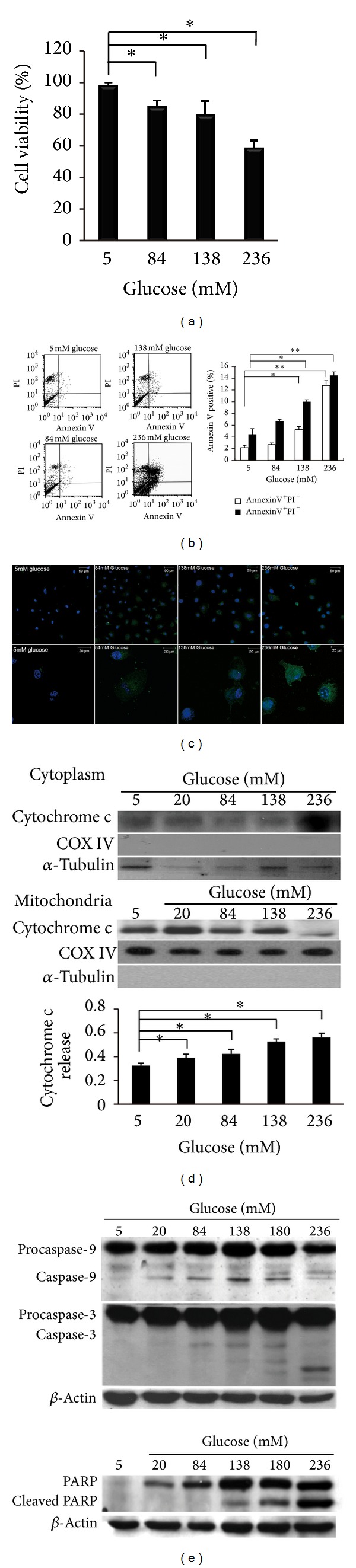
High-dialysate-glucose-induced mitochondrial dependent apoptosis in HPMCs. The HPMCs were isolated from human omentums and cultured at 4 concentrations of glucose, 5 mM (as a control), 84 mM, 138 mM, and 236 mM glucose for 24 h. (a) Exposure to high glucose resulted in a dose-dependent decrease in cellular viability by MTT assay. (b) Apoptotic cells were stained with annexin V-FITC and PI and detected using flow cytometry. (b) High-dialysate-glucose-induced cell apoptosis in both the early apoptotic cell (□, annexin V^+^/PI^−^) and the late apoptotic cells (■, annexin V^+^/PI^+^). (c) HPMCs were stained with annexin V-FITC (green signal) and DAPI (blue signal). Enhanced fluorescent green signals of the annexin V-FITC were observed in the HPMCs treated with high-dialysate glucose. (d-e) Analysis of cytochrome c release, caspase activation, and PARP cleavage of the high-glucose-treated HPMCs. Dose-dependent stimulation of cytochrome c released from the mitochondria into the cytosol was observed in the glucose-treated HPMCs (d). An increase in the cleavage of caspase-3 and caspase-9, and PARP was observed in the high-glucose-treated HPMCs (e). Plots represent the mean ± standard deviation (SD) of 4 independent experiments. Statistical significance: *P < 0.05 compared with the control; COXIV, cytochrome c oxidase subunit IV; PARP, poly(ADP-ribose) polymerase.

The currently used peritoneal dialysates (Dianeal, Baxter) contain 1.5%, 2.5%, and 4.25% dextrose, which are equivalent to glucose concentrations of 84 mM, 138 mM, and 236 mM, respectively. High-dialysate glucose exerted adverse effects on cell viability. Cell viability was observed to be reduced in a dose-dependent manner (*P* < 0.01) (Figure 1(a)). To measure apoptosis, annexin V and PI dual staining was used. A sample scatter graph is shown in Figure 1(b). Mean fluorescence intensities of each channel were plotted as histograms. Based on the fluorescent intensity of each channel, we analyzed the cell population in annexin V^+^/PI^−^ (early apoptotic) and annexin V^+^/PI^+^ (late apoptotic) cells. A dose-dependent increase of early and late apoptosis was observed in the high-dialysate-glucose-treated HPMCs (*P* < 0.05). To visualize the apoptotic features, HPMCs were stained with annexin V-FITC (green signal) and DAPI (blue signal). In the fluorescent images, weak positive signal from annexin V-FITC and PI staining was detected in the HPMCs treated with 5 mM dialysate glucose. Striking fluorescent green signals of the annexin V-FITC were visualized in the HPMCs treated with 238 mM glucose, and shrunken cells were observed in the 238 mM group (Figure 1(c)).

We subsequently determined whether the cytochrome c release from mitochondria and caspase activation was involved in the apoptotic pathway. The localization of cytochrome c in cytosolic and mitochondrial fractions was detected by performing western blotting. COXIV and *α*-tubulin served as controls for the equal loading of proteins and indicated the purity of each fraction (*α*-tubulin for the cytosolic fraction and COXIV for the mitochondrial fraction). We observed a dose-dependent induction of cytochrome c release in the HPMCs stimulated with high levels of glucose (Figure 1(d)). Furthermore, the increased active forms of caspase-3 and caspase-9 were detected in response to the increasing level of glucose. We also observed enhanced cleavage of the PARP in the high-dialysate-glucose-treated HPMCs (Figure 1(e)). These results indicated that high-dialysate-glucose-induced HPMC apoptosis occurred through cytochrome c release and caspase activation.

### 3.2. High-Dialysate-Glucose-Induced Generation of Reactive Oxygen Species, Lipid Peroxidation, and 8-Hydroxy-2′-deoxyguanosine

**Figure 2:**
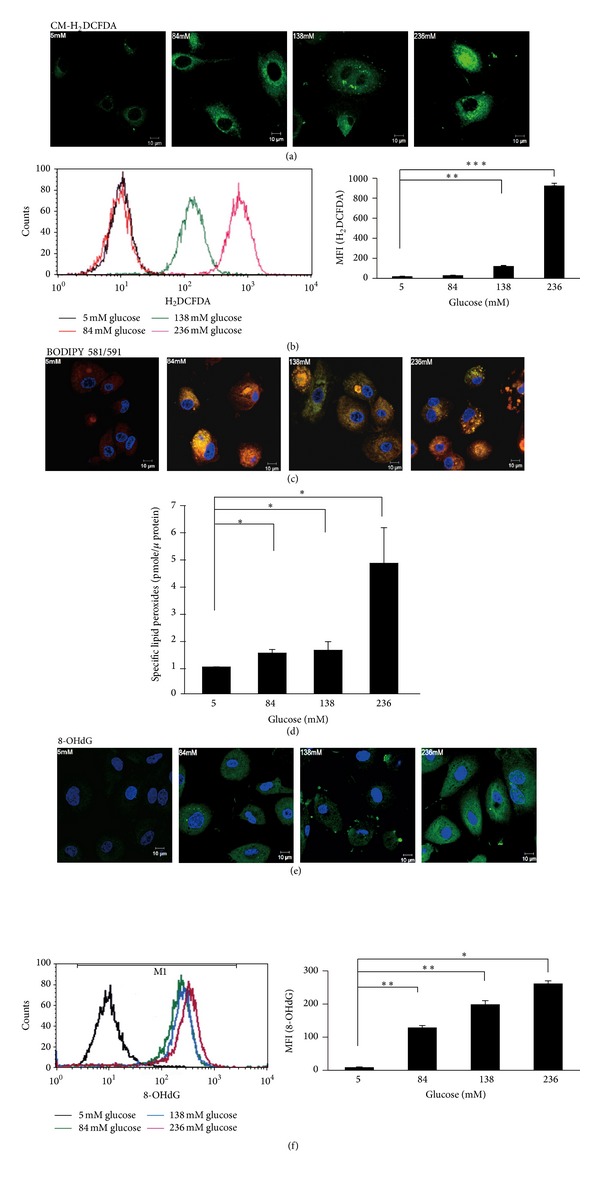
High-dialysate-glucose-induced ROS generation and oxidative damage in HPMCs. (a) To detect ROS generation, CM-H_2_DCFDA staining (green) was performed on the glucose-treated HPMCs. (b) The levels of ROS generation were analyzed using flow cytometry. Exposure to high glucose resulted in a dose-dependent increase in ROS generation. (c) Lipid peroxides were visualized using C11-BODIPY^581/591^ fluorescent staining. After reaction with ROS or free radicals, the red fluorescence of this fluorophore shifted to green. An enhanced green fluorescence intensity was observed in the HPMCs treated with 236 mM glucose. (d) Lipid peroxides were evaluated as TBA-reacted MDA using a HPLC-fluorospectrophotometry method. (e) By staining with an anti-8-OHdG antibody conjugated with FITC, the accumulation and localization of 8-OH-dG were visualized through confocal imaging. Strong green fluorescent images were observed in the mitochondria of the high-glucose-treated HPMCs. (f) The level of 8-OH-dG was measured using flow cytometry analysis. Plots represent the mean ± SD from 4 independent experiments. Statistical significance: *P < 0.05 and **P < 0.01 compared with the control.

To determine whether high-dialysate glucose induces oxidative insults, we analyzed H_2_O_2_ production by performing CM-H_2_DCFDA staining, lipid peroxide levels by performing C11-BODIPY^581/591^ staining, and 8-OHdG levels by staining the cells with an anti-8-OHdG antibody that was conjugated with FITC. The fluorescent signal in the cells was measured immediately using a confocal microscope (Figures 2(a), 2(c), and 2(e)) and quantified using flow cytometry (Figures 2(b) and 2(f)) and HPLC (Figure 2(d)). After exposure to high glucose levels (138 mM and 236 mM), the production of hydrogen peroxide increased nearly to 6.2- and 46.3-fold compared with control group, respectively (Figure 2(b)). Similar results of lipid peroxidation and DNA base damage were observed in glucose-treated HPMCs. C11-BODIPY^581/591^ is a fluorescent dye for indexing lipid peroxidation. After high-dialysate glucose treatments, the fluorescence shifted from red to green in the cytosol, indicating high lipid peroxide levels in the HPMCs (Figure 2(c)). In addition, we determined the MDA level by using HPLC to quantify the level of lipid peroxidation in HPMCs. The indicated glucose treatments induced an approximately 4.9-fold increase of MDA after 238 mM dialysate glucose stimulation (Figure 2(d)). Furthermore, increased 8-OHdG immunopositivity was detected in the mitochondria (Figure 2(e)). An approximate 21.4-fold elevation of 8-OHdG was observed in HPMCs treated with 238 mM glucose (Figure 2(f)). Exposure to high glucose PD solutions induced ROS production and increased oxidative stress and damage in HPMCs.

### 3.3. High-Dialysate-Glucose-Induced Mitochondrial Anomalies

**Figure 3:**
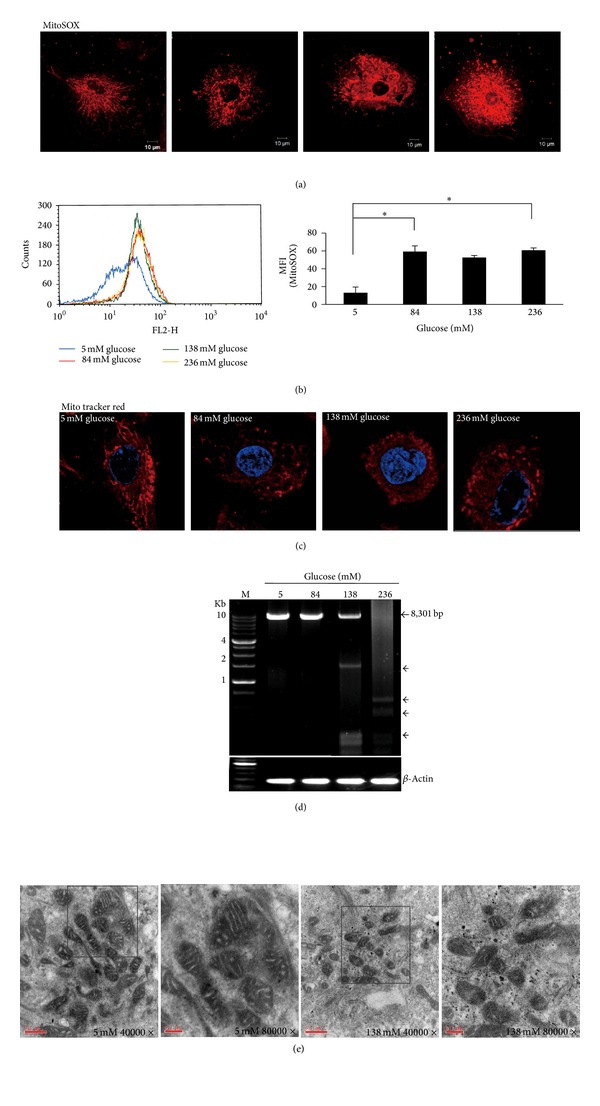
High-dialysate-glucose-induced mitochondrial anomalies. (a,b) A fluoroprobe, MitoSOX Red (MitoSOX), was introduced for the selective detection of superoxides in the mitochondria of HPMCs and was measured using confocal microscopy and flow cytometry. The presence of fragmented mitochondria was prominent in the high-glucose-treated HPMCs. (b) A dose-dependent increase in mitochondrial superoxide production was observed in the high-glucose-treated HPMCs. (c) The shift of mitochondrial morphology from tubular to fragmented forms was observed in response to high-dialysate glucose stimulation. (d) Large-scale deletions of mtDNA were observed using long-range PCR in HPMCs stimulated by 5, 84, 138, and 236 mM glucose. Using the primer sets L8150/H16450, we detected large-scale deletions of mtDNA in HPMCs. The 8301 bp band was amplified from the wild-type mtDNA. Multiple mtDNA deletions (*◄*) were amplified from the HPMCs treated with 138 and 236 mM glucose. Lane M, 1 Kb DNA ladder. (e) The ultrastructure of mitochondria in HPMCs was observed using a transmission electron microscope. These mitochondria exhibited arch-shaped cristae and vacuolization in high-dialysate-glucose-treated HPMCs. Original magnification ×4000 and ×8000. The higher magnification field (×8000) is indicated by the square in the ×4000 image. Plots represent the mean ± SD from 4 independent experiments. Statistical significance: *P < 0.05 and **P < 0.01 compared with the control.

Because 8-OHdG accumulated considerably in mitochondria, we analyzed the effect of high-dialysate glucose on mitochondria. MitoSOX Red (MitoSOX) staining was used to measure mitochondrial superoxides level (Figure 3(a)). An approximate 4.5-fold increase in mitochondrial superoxides was detected in the HPMCs treated with 84 mM dialysate glucose (Figure 3(b)). In addition, we observed that the ROS-induced oxidative damage caused mitochondrial fragmentation (Figures 3(a) and 3(c)). Using a long-range PCR technique, we screened large-scale deletions of mtDNA in dialysate-glucose-treated HPMCs. We detected large-scale deletions of mtDNA in HPMCs by using the primer sets L8150/H16450. The 8301-bp PCR products were generated from wild-type mtDNA. Multiple large-scale deletions of mtDNA were detected in the 138 mM and 236 mM dialysate-glucose-treated HPMCs (Figure 3(d)). Regarding changes in mitochondrial ultrastructure, TEM images showed that high-dialysate glucose caused a reduction of the size of mitochondria and most mitochondria exhibited peripheral arch-like cristae (Figure 3(e)).

### 3.4. Antioxidant N-Acetylcysteine Attenuated High-Dialysate-Glucose-Induced Oxidative Insults and Mitochondrial Fragmentation

**Figure 4:**
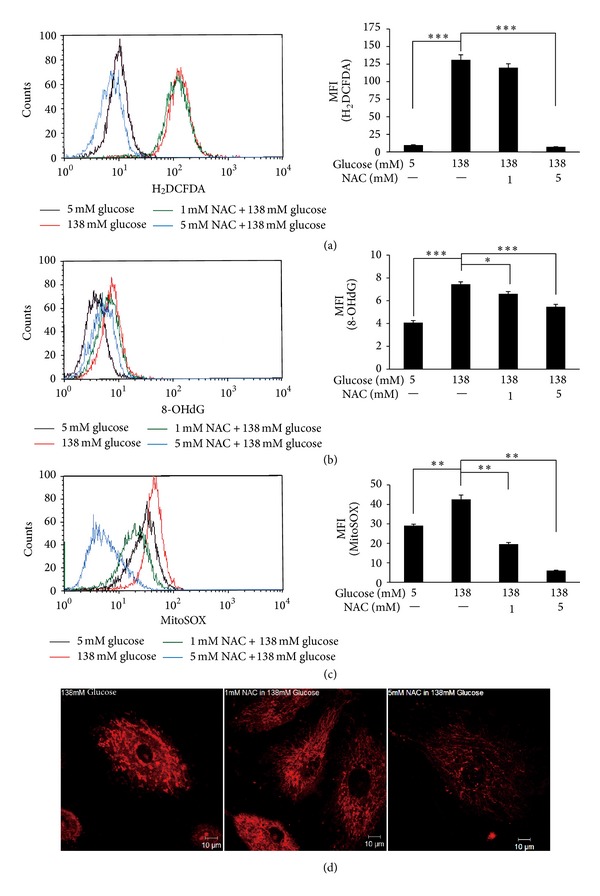
NAC, an ROS scavenger, reduced high-dialysate-glucose-induced ROS generation and oxidative damage in HPMCs. HPMCs were pretreated with 1 or 5 mM NAC for 1 h before glucose stimulation. The levels of cellular ROS (a), lipid peroxides (b), and mitochondrial superoxides (c) were detected using flow cytometry. Pretreatment with 5 mM NAC reduced high-dialysate-glucose-induced oxidative insults. (d) The fluorescent intensity of mitochondrial superoxide and mitochondrial morphology using MitoSOX Red were detected by confocal microscopy. Antioxidant NAC reduced mitochondrial superoxide generation and rescued mitochondria from extensive fragmentations.

NAC, an antioxidant, acts as a cysteine donor and a precursor of glutathione synthesis and contributes to improving free radical surveillance and mitochondrial function. HPMCs were pretreated with 1 or 5 mM NAC for 1 h before glucose stimulation. After dialysate glucose stimulation, pretreating HPMCs with 5 mM NAC reduced the production of hydrogen peroxide (*P* < 0.001), 8-OHdG (*P* < 0.001), and mitochondrial superoxides (*P* < 0.01) compared with the 138 mM dialysate glucose group that was not pretreated with NAC (Figures 4(a), 4(b), and 4(c)). Antioxidant NAC rescued mitochondria from extensive fragmentations in a dose-dependent manner (Figure 4(d)).

### 3.5. N-Acetylcysteine Recovered Mitochondrial Membrane Potential and Reduced Apoptosis in High-Dialysate-Glucose-Treated Human Peritoneal Mesothelial Cells

**Figure 5:**
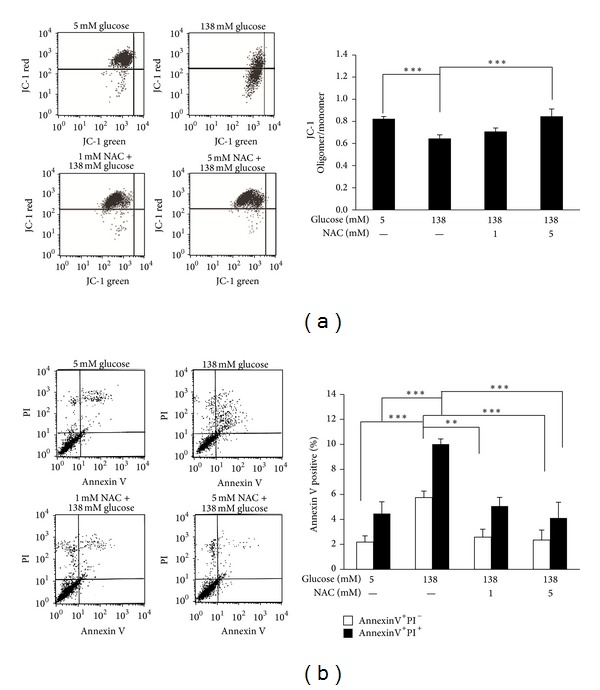
NAC attenuated high-dialysate-glucose-induced mitochondrial dysfunction and apoptosis. (a) A dot plot of mitochondrial membrane potential determined using flow cytometry is shown in 4 groups (5 mM glucose, 138 mM glucose, 1 mM NAC with 138 mM glucose, and 5 mM NAC with 138 mM glucose). The mitochondrial membrane was stained with JC-1, existing as a green fluorescent monomer at low membrane potentials and as orange fluorescent aggregates at high membrane potentials. (b) Histograms represent the JC-1-oligomer oligomer/monomer (green) fluorescence ratio. (c) A dot plot of cell apoptosis determined using flow cytometry is shown. The 4 groups were obtained and the relative percentages of cells at the various apoptotic stages are presented. The lower-left field indicates viable cells, the lower-right field indicates early apoptotic cells (annexin V positive and PI negative), and the upper-right field indicates late apoptotic cells (annexin V positive and PI positive). (b) Flow data are presented for 3 experiments, in which apoptosis was induced through high-dialysate glucose stimulation. Pretreatment of NAC reduced high-dialysate-glucose-induced apoptosis.

Mitochondrial membrane potential is an indicator of mitochondrial function and a sensitive index of cell damage because it is easily influenced by environmental stress. Following 138 mM dialysate glucose treatment, JC-1 accumulated preferentially in mitochondria, existing as green fluorescent monomer at low membrane potentials. NAC treatment caused mitochondrial membrane potential to recovery (*P* < 0.01, Figure 5(a)) and prevented the HPMCs from undergoing high-dialysate-glucose-induced cell apoptosis (Figure 5(b)).

## 4. Discussion

Increasing evidence has indicated the crucial role of HPMCs in maintaining the functional integrity of the peritoneal membrane [14]. Impairment of this membrane caused by long-term exposure to dialysate can lead to peritoneal fibrosis, ultrafiltration failure, degenerative changes, including mesothelial cell loss, the accumulation of extracellular matrices, and vasculopathy [5]. In this study, we demonstrated that ROS overproduction occurred in HPMCs after exposure to high-dialysate glucose, which impaired mitochondrial bioenergetics and caused mtDNA instability, leading to apoptotic cell death in HPMCs. Antioxidant NAC protects HPMCs from high-dialysate-glucose-induced apoptosis by reducing oxidative stress and ameliorating the dissipation of mitochondrial membrane potential.

The hallmarks of apoptosis are DNA fragmentation and caspase activation, which lead to PARP cleavage. In our study, high-dialysate-glucose-induced apoptosis occurred through the release of cytochrome c, sequential caspase-9, and caspase-3 activation, as well as PARP cleavage. PARP, a nuclear enzyme involved in DNA repair, has been identified as a substrate for caspase-3 cleavage and has been observed to be cleaved into 24 and 89 kDa fragments that contain the DNA-binding domain and the active site of PARP, respectively, during late stages of apoptosis in various cells [15].

ROS were produced mainly by an electron transport chain of mitochondria as a by-product of oxidative phosphorylation. When glucose levels rise, enhanced glycolysis is accompanied by increased pyruvate supply to mitochondria, stimulating mitochondrial respiration, which was accompanied by a simultaneous rise in oxidative stress. Cumulative evidence supports a pivotal role of mitochondria in the excess metabolic demand of high glucose. A previous study indicated that an increased mtDNA copy number responds to high levels of glucose in human mesangial cells, thereby increasing mitochondrial superoxides [16]. In another study, mitochondrial respiratory complex III activity was inhibited and the production of mitochondrial superoxides increased [17]. It is proposed that excessive glucose metabolism generates ROS, exceeding the defense mechanisms of HPMC mitochondria responding to high-dialysate glucose, thus forming a vicious cycle that causes electron transport chain (ETC) uncoupling and increases proton leak in the ETC.

In our study, a markedly increased production of hydrogen peroxide and 8-OHdG was detected in 238 mM high-dialysate-glucose-treated cells, and hydrogen peroxide and 8-OHdG particularly accumulated in the HPMC mitochondria. High-dialysate glucose is suggested to impair cell function through lipid peroxidation, alter membrane fluidity and integrity, and perturb the structure and morphology of mitochondria [18]. In addition, high-dialysate glucose induces mutagenesis through 8-OHdG by its miscoding properties: instead of dCMP, dAMP can be incorporated opposite 8-OHdG during replication leading to G→T nucleotide substitution [19]. If not repaired, 8-OHdG modifications in DNA are mutagenic and can lead to increased mtDNA deletion and cause mitochondrial genome instability [20]. The deletion region encompasses the genes in mitochondrial respiratory complexes I, III, IV, and V and might contribute to the cause of ATP depletion. Increased lipid and protein modification through hyperglycemia-induced oxidative stress causes diabetic complications [16]. The oxidation products of lipids, DNA, and proteins, such as MDA, thiobarbituric acid reactive substances, 8-OHdG, advanced oxidation protein products, and protein carbonyl, can act as circulating biomarkers of the susceptibility to developing diabetic complications [21]. Oxidative insults cause oxidative damage to mtDNA, elicit multiple mtDNA deletions, and interfere with the synthesis of certain protein complexes in the ETC.

This study is the first to demonstrate that HPMCs undergo mitochondrial fragmentation after exposure to high-dialysate glucose. The mitochondria exhibited a long tubular shape in 5 mM glucose treatment but became shorter and smaller and even fragmented after exposure to high-dialysate glucose (Figures 3(b) and 3(c)). Mitochondria are dynamic organelles, undergoing constant fission and fusion regulated by machinery involving large GTPase family proteins and their regulators [22]. Perturbation of the dynamic balance between mitochondrial fusion and fission results in mitochondrial fragmentation, leading to impairment of oxidative phosphorylation [23]. Mitochondrial fragmentation has been demonstrated to be a common feature in stress-induced apoptosis. During apoptosis, mitochondria are extensively fragmented; this fragmentation is associated with MPT pore opening and causes cytochrome c and apoptotic effectors to leak, triggering caspase activation and subsequent apoptosis [24–26]. The inhibition of mitochondrial fragmentation in numerous mammalian cell lines results in reduced cytochrome c release to the cytosol and inhibition of apoptosis [25].

Mitochondrial membrane potential is used for monitoring changes in mitochondrial physiologic activity because it relates to the capacity of oxidative phosphorylation. The results of our study indicated that NAC treatment reduced ROS generation and ameliorated the dissipation of mitochondrial membrane potential caused by high-dialysate glucose. The loss of mitochondrial membrane potential is related to the opening of the MPT pore [11]. This, in turn, activates a series of proapoptotic factors leading to apoptosis in the cell. An excessive number of ROS are the direct cause of mitochondrial dysfunction because reducing the ROS levels by using NAC in high-dialysate glucose incubation prevents the dissipation of Δ*ψ*m and subsequent apoptosis of HPMCs.

Hemodialysis (HD) patients have been shown to have elevated oxidative stress compared with healthy matched controls. This is postulated as contributing to the high risks of cardiovascular disease, acute-phase inflammation, and mortality in patients undergoing hemodialysis therapy [27, 28]. Antioxidants have been used as therapies to decrease oxidative stress and improve CVD morbidity in HD patients and provide no beneficial effects [27, 28]. Three such studies reported a positive therapeutic effect; alpha-tocopherol [29], N-acetylcysteine [30], and combined vitamins C and E [31] were used, respectively, thus providing a useful and therapeutic option in PD patients [31]. In the future, population-based longitudinal studies are needed to predict CVD outcomes in HD patients. Further study is urgent to establish clinically accepted and validated biomarkers for oxidative stress to validate the therapeutic effects. The time-course and dosing studies of antioxidant therapy should be tested to ensure that the reduction in oxidative stress is achieved.

In summary, high-dialysate-glucose-induced HPMC apoptosis is directly caused by the generation of ROS through injury to the mitochondrial network, as well as mitochondrial dysfunction. Large-scale mitochondrial deletions and mitochondrial fragmentation concomitantly contributed to impairment of mitochondrial metabolism, triggering further ROS generation and inducing HPMC apoptosis. NAC reduced mitochondrial fragmentation and ameliorated HPMC apoptosis through an antioxidative effect. This approach to suppressing ROS overproduction and preventing mitochondrial fragmentation has potential in retarding the development and progression of the long-term complications of dialysis.
